# Performances of Polymer-Dispersed Liquid Crystal Films for Smart Glass Applications

**DOI:** 10.3390/polym15163420

**Published:** 2023-08-16

**Authors:** Muhammad Shahriyar Islam, Kah-Yoong Chan, Gregory Soon How Thien, Pei-Ling Low, Chu-Liang Lee, Sew Kin Wong, Ervina Efzan Mhd Noor, Benedict Wen-Cheun Au, Zi-Neng Ng

**Affiliations:** 1 Centre for Advanced Devices and Systems, Faculty of Engineering, Multimedia University, Persiaran Multimedia, Cyberjaya 63100, Selangor, Malaysia; mshahriyar98@gmail.com (M.S.I.); gregory@mmu.edu.my (G.S.H.T.); pllow@mmu.edu.my (P.-L.L.); cllee@mmu.edu.my (C.-L.L.); skwong@mmu.edu.my (S.K.W.); 2 Centre for Manufacturing and Environmental Sustainability, Faculty of Engineering and Technology, Multimedia University, Jalan Ayer Keroh Lama, Bukit Beruang, Melaka 75450, Malaysia; ervina.noor@mmu.edu.my; 3 Sri Desa International Secondary School, Taman Desa, Kuala Lumpur 58100, Malaysia; benedict.au@sridesa.edu.my; 4 School of Electrical Engineering and Artificial Intelligence, Xiamen University Malaysia, Jalan Sunsuria, Bandar Sunsuria, Sepang 43900, Selangor, Malaysia; zineng25@gmail.com

**Keywords:** polymer-dispersed liquid crystals, visible light transmittance, ultraviolet rejection, infrared rejection, current consumption, apparent power consumption

## Abstract

Polymer-dispersed liquid crystal (PDLC) film is an active smart film penetrating the market due to its unique functionalities. These functional characteristics include switchable tint capabilities, which shield building residents from the sun’s harmful ultraviolet (UV) rays, improve energy-saving features, and produce higher cost-efficiency. Although PDLC films are promising in several applications, there is still ambiguity on the performance of PDLC films. Particularly, the sizing effects’ (such as film thickness and area) correlation with visible light transmission (VLT), ultraviolet rejection (UVR), infrared rejection (IRR), light intensity, current consumption, and apparent power consumption is not well understood. Therefore, this study investigated the sizing effects of PDLC films, including the thickness effect on VLT, UVR, IRR, light intensity, and area influence on current and apparent power consumptions. The varying applied voltage effect on the light transmittance of the PDLC film was also effectively demonstrated. A 0.1 mm PDLC film was successfully presented as a cost-efficient film with optimal parameters. Consequently, this study paves the way for a clearer understanding of PDLC films (behavior and sizing effects) in implementing economic PDLC films for large-scale adoption in commercial and residential premises.

## 1. Introduction

Smart glass, also known as switchable glass, can reduce the amount of light penetrating it by changing its opacity [1]. Thus, smart glass has emerged as a groundbreaking and innovative alternative to traditional windows due to its ability to shield sunlight. Smart glass can be categorized into passive or active technologies [2,3]. These smart glasses respond to either non-electrical stimuli (passive) or require electrical voltage stimuli to operate (active). With the maturity of the internet of things (IoT), active smart glasses are being incorporated into the wireless-connected era [4]. Hence, smart devices, such as smartphones, can control the opacity of active smart glasses. Among the variations in smart glasses, polymer-dispersed liquid crystal (PDLC) is one of the most popular smart glasses due to its widespread applications [5]. Generally, a PDLC film is attached to the window for commercial applications on building fenestrations.

The PDLC film’s opacity depends on the liquid crystals’ alignment inside the film [6]. These crystals are randomly aligned without an applied voltage, making the glass opaque. When a voltage is successfully applied, the crystals align along the direction of the electric field, allowing light to penetrate through and causing the glass to become transparent. The phenomenon of PDLC’s working principle gave rise to its attractive unique properties, such as faster switching speed (in milliseconds), higher privacy, and lower power consumption [7].

The structure of PDLC films consists of several layers, and the innermost layer is a continuous polymer matrix. Subsequently, the polymer matrix contains holes filled with micro-sized liquid crystals (LC). These LC are responsible for controlling the opacity of the PDLC films [8]. Depending on the orientation of the LC, the incident light can either penetrate through the film or be reflected. The LC droplets and polymer matrix are usually mixed to create a homogeneous solution. This solution is held in place by sandwiching it between two thin layers of indium tin oxide (ITO). Since the ITO layers act as electrodes for the structure, applying an electrical voltage to the ITO will transfer the electrical current into the polymer solution. Therefore, this process causes the LC to reorient themselves in the direction of the electrical field. Finally, the outermost layer of the PDLC film is the polyethylene terephthalate (PET) layer; PET acts as a protective layer for the structure to prevent the PDLC film from being damaged or scratched [9].

Numerous studies are being conducted on different aspects of the PDLC films, such as enhancing the electro-optical properties of the PDLC film for implementation into various applications and incorporating IoT features into the smart film. J.Hu et al. fabricated PDLC films using two different monomers called hydroxyl methacrylate and fluorinated methacrylate [10]. It was observed that the fabricated film had an improved contrast ratio and low driving voltage [10]. Z. Shi et al. prepared PDLC films using a mixture consisting of nanofiber films, which greatly improved the optoelectronic and fluorescence properties of their film [11]. On the other hand, Z. He et al. optimized the properties of their PDLC film by applying a pre-orientation voltage during the polymerization [12]. This caused the driving voltage of the film to reduce drastically to a safe level [12]. Furthermore, M.S. Islam et al. developed an IoT smart controller, which was capable of operating the PDLC films wirelessly and without any human interaction [13]. This would allow for the film to be implemented for a wider range of applications, such as office meeting rooms, buildings, transportation, hospital rooms, and more [13]. Moreover, A. Abosaq et al. designed a miniature greenhouse system using an Arduino microcontroller that incorporates commercial PDLC films [14]. The role of the film is to control the amount of sunlight penetrating through the glass windows [14].

Currently, the methods used by researchers to fabricate the smart film are solution-induced phase separation (SIPS) [15], thermally induced phase separation (TIPS) [16,17], and polymerization-induced phase separation (PIPS) [18,19]. In recent years, some glass companies have been working on ways to implement PDLC film as an interactive projection screen. Furthermore, IoT is also being integrated with PDLC films to allow for wireless switching of the film using either remote control or mobile applications.

Apart from incorporating LC into PDLC film, researchers are also implementing LC into a wide range of applications. G.F. Sung fabricated a cholesteric liquid crystals (CLCs) device that acted as a smart glass [20]. The transparency of the smart glass can be controlled either manually by human interaction or autonomously by detecting the environmental temperature [20]. Liquid crystals can also be found in transparent displays, such as liquid crystal displays (LCD), due to the light-modulating properties of the crystals [21]. As voltage is applied to an LCD display, the liquid crystals within it change into the micrometer-sized polydomain state [21]. This causes the incident light to be scattered out of the display creating images on the screen [21]. Another application of liquid crystals is in light modulators. W.F. Chiang et al. fabricated a liquid crystal elastomer (LCE) film with a terahertz metamaterial deposited on it [22]. This fabricated film acts as an optically tunable intensity modulator, which can be used for terahertz communication and imaging [22]. Additionally, liquid crystals are also used in switchable smart windows due to their ability to change opacity based on the voltage frequency that is applied. The type of liquid crystals that are widely used in smart windows are dual-frequency liquid crystals (DFLCs) [23]. The crystals scatter light as the frequency of the applied voltage is close to its crossover [23]. Furthermore, L. Ma et al. reported the usage of liquid crystals in electro-optical switches [24]. They studied the switching time of chiral liquid crystals with 1D photonic microstructures [24]. It was observed that, as voltage was applied to the structure, the orientation of the molecules was altered rather than the direction of the liquid crystals, which caused the switching time to be as short as 100 ns [24]. Moreover, optical grating is another component that utilizes liquid crystals to diffract light into several beams [25]. This can be achieved by fabricating optical gratings based on PDLC cells with in-plane switching electrodes [25]. The electrodes in this structure are patterned; hence, it is able to generate an electric field, which can control the orientation of the liquid crystals [25]. Thus, a spatial periodic distribution is created, which causes the formation of the optical grating [25]. LC in the form of polymer-stabilized liquid crystals (PSLCs) can also be used to fabricate micro lenses [26]. J.B. Wu reported developing an electrically tunable micro lens with polymer stabilization, which involves doping the LC mixture with RM257 reactive monomer and photo-initializer [26]. Another application of liquid crystals is in special light field generators [27]. C.T. Xu et al. illustrate how planar optics are able to modulate light with the use of polymer-stabilized cholesteric liquid crystals [27].

Despite the wide implementation of the PDLC film for various real-life applications, there is still a lack of understanding of the performance of the PDLC film in terms of its energy consumption and transmittance [28]. Hence, research studies are being performed on these technical parameters to find the most efficient and cost-saving method to implement PDLC films as a light-filtration system [29]. A clearer understanding is necessary for investigating the electro-optical properties of PDLC films and their correlation to the different dimensions of PDLC film. This information is vital as various film sizes are currently available for purchase on the market. In the commercial market, a PDLC film’s price is determined by its performance and dimensions. For example, a 1 m^2^ size PDLC film costs between $85 and $130, while bigger dimensions have an even higher cost. Moreover, the PDLC film installation for commercial and building premises requires large film quantities, greatly increasing the cost [30,31]. Thus, understanding the performance of the PDLC film with sizing effects will allow for a more economical approach to be adopted.

This study aimed to investigate the sizing effects of the PDLC film corresponding to the performance of the PDLC film, particularly the visible light transmittance (VLT), ultraviolet rejection (UVR), infrared rejection (IRR), light intensity, and current and apparent power consumptions. Various thicknesses and areas of the PDLC film were investigated, and the optimal parameters were effectively understood. This study acquired a clearer framework for selecting the right PDLC film in the smart glass industry.

## 2. Working Principle and Preparation Process

Within PDLC films, liquid crystals are usually entrapped inside a transparent polymer medium. This causes the formation of micrometer-sized liquid crystal droplets. The liquid crystal droplets are generally oriented in random directions in the polymer matrix. Thus, as incident light hits the PDLC film, the light is scattered as there is a mismatch in the refractive index of the polymer matrix and liquid crystal droplets. Hence, the PDLC film appears opaque.

When an external electric field is applied to the PDLC film, the liquid crystal droplets reorientate themselves towards the direction of the field. This causes the refractive index of the polymer matrix and liquid crystals to match; thus, the PDLC film changes its opacity from opaque to transparent.

**Figure 1:**
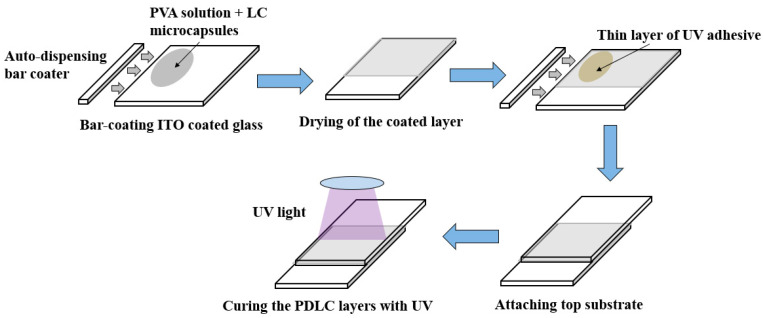
Fabrication process of PDLC films [32].

The process of fabricating PDLC films is shown in Figure 1 below. First, substrates of indium tin oxide (ITO) are cleaned with deionized water, acetone, and isopropyl alcohol (IPA) [32]. Next, a solution of polyvinyl alcohol (PVA) and LC microcapsules is poured onto the surface of the ITO [32]. The solution is then bar-coated to ensure an even distribution onto the substrate [32]. Once the solution has been bar-coated and dried, a thin layer of adhesive is deposited on top of the dried solution [32]. Then, another ITO-coated glass substrate is placed on top of the adhesive [32]. To ensure the substrates are permanently sandwiched together, UV light is shone onto the substrates. The end result is a PDLC device that has been fabricated [32].

## 3. Materials and Methods

**Figure 2:**
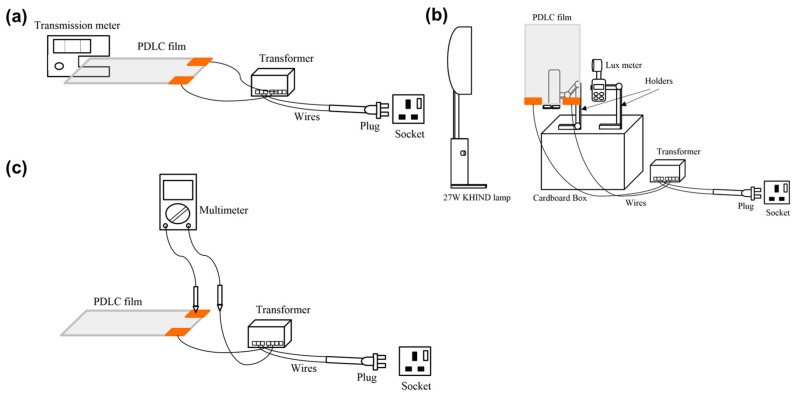
(**a**) Schematic diagram indicating the VLT, UVR, and IRR measurements using an LS162 transmission meter. (**b**) Schematic diagram indicating the setup to measure the light intensity through a PDLC film. (**c**) Schematic diagram indicating the setup to measure current consumption through a PDLC film.

This study investigated the VLT, UVR, IRR, light intensity, and current and apparent power consumptions for PDLC films. The PDLC films that were used in these experiments are commercially fabricated PDLC films with fixed dimensions and thickness. The dimension sizes of the PDLC films are listed in Table 1. Meanwhile, the measurement setup to characterize the performance of the PDLC films is depicted in Figure 2.

### 3.1. Measuring VLT, UVR, and IRR for PDLC Films

Figure 2a shows the method used to measure the transmittance values. Initially, a PDLC film was used to perform the VLT, UVR, and IRR measurements using an LS162 transmission meter. The transmission meter used in this experiment is a split-beam spectrometer that can measure UV, IR, and visible light peak lengths of 365 nm, 940 nm, and 380–760 nm, respectively [33]. The range of measurement of the device is 0–100% [33]. A 30 W step-down transformer with a 60 VAC output was then connected to the film’s busbar during the study to alter the transparency of the film. Finally, this procedure was repeated for all the remaining PDLC films.

### 3.2. Measuring Light Intensity for PDLC Films

The light intensity measurements were performed on all PDLC films (see Figure 2b). Initially, a 27 W fluorescent lamp held at a fixed distance from the PDLC films was applied as the light source. A holder was then kept behind the PDLC films to place the UT383 lux meter, which was responsible for measuring the light intensity passing through the PDLC films. Throughout the experiment, a step-down transformer was connected to the film’s busbar to control the film’s opacity during the measurement process. Furthermore, the investigation was conducted in a completely dark room to avoid exposure to external light sources. Measurements were taken by pointing the light source at a PDLC film and measuring the light intensity with the lux meter. This procedure was repeated for different film thicknesses in their opaque and transparent states.

### 3.3. Measuring Current and Apparent Power Consumptions for PDLC Films

The films of similar thickness and different areas were utilized in this part of the study to obtain the relation between current consumption and the PDLC films’ sizing. First, the PDLC film areas were 210 mm × 75 mm, 210 mm × 150 mm, 210 mm × 297 mm, and 420 mm × 297 mm, while the film thickness was kept constant at 0.394 mm. For measuring the current consumption, a digital multimeter was used. The digital multimeter used to measure the voltage and current was DT2905A. This device has a voltage measurement error rate and current measurement error rate of 0.5% and 1.2%, respectively [34]. Before the measurement, the transformer was switched on to allow the current to pass through the film. The amount of current drawn by the PDLC films was then recorded using a multimeter (see Figure 2c). Subsequently, the obtained current consumption values were inserted into Equation (1) to calculate the apparent power consumption of the PDLC films with different areas. The obtained current consumption values were utilized using Equation (1) [31]. Thus, the apparent power consumption of all PDLC films with different areas is calculated as follows:

(1) S=V×A

where *S* is the apparent power consumption, *V* is the supplied voltage, and *A* is the current consumption.

## 4. Results and Discussions

### 4.1. Thickness Influence on the Performance of PDLC Films

**Figure 3:**
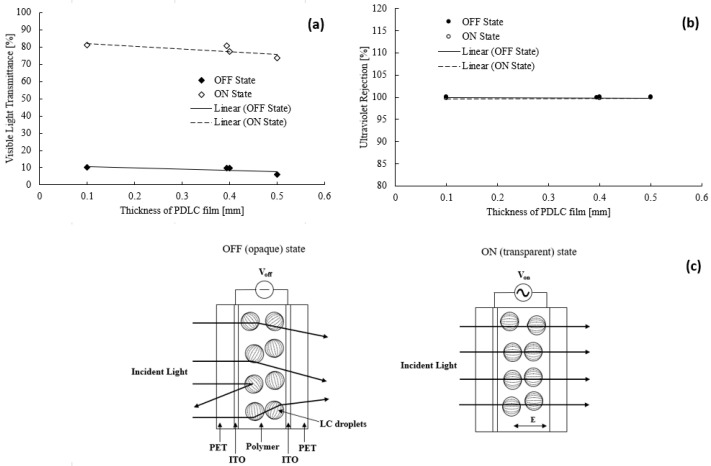
(**a**) Visible light transmittance and (**b**) ultraviolet measurements for PDLC films with thicknesses ranging from 0.1 to 0.5 mm. (**c**) Operation mechanism of a PDLC film in its OFF (opaque) and ON (transparent) states.

Two experimental sets were performed to obtain the value of the technical parameters (VLT, UVR, IRR, and light intensity) influenced by the thickness of the PDLC film. Figure 3a illustrates the thickness influence on the VLT of the PDLC film. Based on the trend of the figure, the VLT value reduced when the thickness of the film gradually increased from 0.1 to 0.5 mm. A significant VLT difference was observed for the PDLC film’s opaque and transparent states. The highest and lowest recorded VLTs in the OFF state were 10.2% (thickness = 0.1 mm) and 6.0% (thickness = 0.5 mm), respectively. Alternatively, the highest VLT value obtained in the ON state was 81.2% at 0.1 mm, while the lowest VLT value in the ON state was 73.7% at 0.5 mm.

The decrease in VLT with increasing film thickness is due to the scattering of the incident light that occurs within the film [35]. The scattering is caused by the number of liquid crystal droplets present in the film [35]. In thin PDLC film, there is a lower quantity of liquid crystal droplets [35]. Therefore, when the visible light passes through the film, the incident light is scattered relatively little, resulting in a higher VLT value [35]. On the other hand, in thicker PDLC films, there is a larger amount of liquid crystal droplets [35]. As a result, the light is scattered off the LC microdroplets more when it passes through the film, which reduces the VLT value [36].

The PDLC film consists of microdroplets that are spread within the polymer matrix due to phase separation. During the OFF and ON states of the PDLC films, the difference in VLT was due to the arrangement of the LC molecules within the films [37]. When switched off, the LC molecules within each microdroplet were arranged randomly (see Figure 3c). Therefore, the polymer matrix’s refractive index does not match the LC droplets. Hence, as the incident light tries to penetrate through the PDLC film, it is scattered in various directions because the light bounces off the LC microdroplets, which are arranged randomly in the film. This causes the VLT value to lower since the transmittance of light decreases. Conversely, the visible light passed through the PDLC films as the LC molecules of the PDLC were aligned when switched on. Consequently, the refractive index of the polymer matrix and the LC droplets matched. This outcome reduced the amount of incident light scattering as a higher percentage of light is able to pass through the gaps within the LC microdroplets, suggesting a higher VLT value [38]. Despite the reduced VLT value with the increased thickness of the PDLC film, the reduced transmittance was insignificant. Therefore, a PDLC film with a thickness of 0.1 mm was sufficient to achieve the required tinting performance. Moreover, the film could achieve a high VLT reduction at a relatively lower cost than a thicker 0.5 mm PDLC film (costing significantly higher).

Figure 3b portrays the ultraviolet (UV) percentage rejected by the PDLC films ranging in thickness from 0.1 to 0.5 mm. The UVR values remained constant despite the increase in the PDLC film’s thickness. This observation was acquired in the ON and OFF states of the films. In addition, the UVR values for the ON and OFF states of the PDLC films were almost identical. The lowest recorded UVR in the ON state was 99.6% (thickness = 0.4 mm), and the highest was 99.8% (thickness = 0.5 mm). In the OFF state, the lowest UVR percentage obtained was 99.8% at 0.394 mm, while the highest UVR percentage was 100% at 0.1, 0.4, and 0.5 mm thicknesses.

The factor responsible for blocking the UV light from the source was the cut-off wavelength of the PDLC films [39]. Since the cut-off wavelength of the PDLC film was in the UV range, the incident light from the source could not pass through the film. This process caused the UV transmission to drop drastically to almost 0%. Interestingly, changing the films’ thickness had a negligible impact on the UVR values. This observation was consistent with that reported by Hemaida et al. [38], in which the UV transmittance for PDLC in the translucent state was only 8% [37]. Based on these findings, thin and thick PDLC films were concluded to produce the same UVR amount. The operability of a thinner PDLC film can protect residents (human skin) of buildings from harmful UV light. Additionally, purchasing a thin PDLC film is much cheaper than a thicker one.

**Figure 4:**
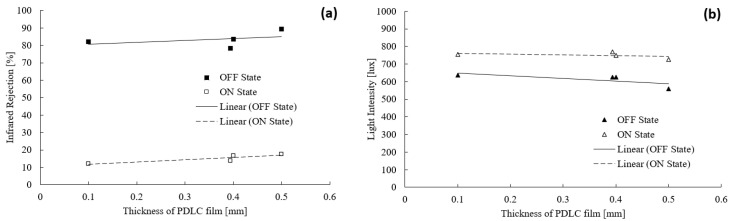
(**a**) Infrared rejection and (**b**) light intensity measurements for PDLC films with thicknesses ranging from 0.1 to 0.5 mm.

Figure 4a presents the infrared light percentage blocked by the PDLC films ranging in thickness from 0.1 to 0.5 mm. As the film thickness increased, the IRR percentage also increased. Thus, the IRR value was higher when the film was in the OFF state than in the ON state. The lowest recorded IRR percentage (ON state) was 12.1% at 0.1 mm, and the highest IRR percentage was 17.6% at 0.5 mm. In the OFF state, the lowest and highest IRR percentages were 78.2% at 0.394 mm and 89.2% at 0.5 mm. The IRR percentages in the OFF and ON states differed by a large margin due to the scattering of the incident infrared light on the PDLC films. In the OFF state, the LC molecules exhibited a random arrangement, resulting in the refractive index of the polymer matrix and the LC droplets mismatching with each other. Hence, this process promoted a greater scattering of infrared light, producing a significantly larger IRR value as a higher proportion of infrared light was scattered [40]. Meanwhile, the polymer matrix and the LC droplets acquired a refractive index that matched as the LC molecules were aligned in the ON state. There was less scattering of the incident infrared light, and more light penetrated through the films, reducing the IRR value.

Due to the dimension of the liquid crystal droplets, the IRR percentage became larger with an increase in the thickness of the PDLC film. As the film was thicker, the LC droplets became larger [41]. Hence, more infrared light scattering occurred, increasing the IRR value. On the contrary, the LC droplet size was smaller, which indicated less scattering and a smaller IRR value as the film thickness decreased. Although the IRR percentage increased as the thickness of the PDLC film increased, the percentage difference between the thin and thick dimensions of the film was still tolerable. The value difference between the ON and OFF states of the film was measured to be 11% and 5.5%, respectively. These percentages were acceptable, suggesting a more cost-effective solution for widespread implementation in thinner PDLC films than thicker films.

The light intensity effect of PDLC film thicknesses from 0.1 to 0.5 mm is depicted in Figure 4b. The overall trend demonstrated that the light intensity decreased when the thickness of the film increased. This outcome was observed for both the ON and OFF states of the film. When switched off, the recorded minimum light intensity was 558 lux at 0.5 mm, and the maximum was 637 lux at 0.1 mm. In the ON state, the highest and lowest recorded light levels were 756 lux at 0.1 mm and 726 lux at 0.5 mm, respectively. As the thickness of the PDLC film increases, there are more microdroplets present within the film. This causes the quantity of LC molecules to increase too. Hence, as incident light tries to pass through the PDLC film, the light is bounced off the LC microdroplets, resulting in a greater amount of light scattering [42].

With the reduced thickness of the PDLC films, there were fewer liquid crystal droplets within the film. Resultantly, less scattering and more incident light penetration were generated, increasing the light intensity. Even though the PDLC film’s thickness increased, the light intensity reduction was minimal. This is due to the type of light that was emitted by the source. In this experiment, a fluorescent lamp was used as the light source. Hence, the majority of the light emitted by the lamp was localized to the visible light region. As shown in Figure 3a, the PDLC film thickness causes insignificant change to the percentage of VLT; thus, a similar result was obtained for the light intensity penetration experiment. In the ON state, the light intensity dropped by only 30 lux from 0.1 mm to 0.5 mm. Likewise, the light intensity was reduced by 79 lux in the OFF state. Hence, the mentioned differences were negligible when compared to the light intensity obtained without any PDLC film, such as 960 lux.

The results recorded in these experiments have been compiled in Table 2 for comparison. Based on the table, it can be observed that purchasing PDLC film with an area of 210 mm × 297 mm with 0.100 mm thickness is the best option since the PDLC film would have a lower cost while having a promisingly high electro-optical performance.

### 4.2. Area Influence on the Performance of PDLC Films

**Figure 5:**
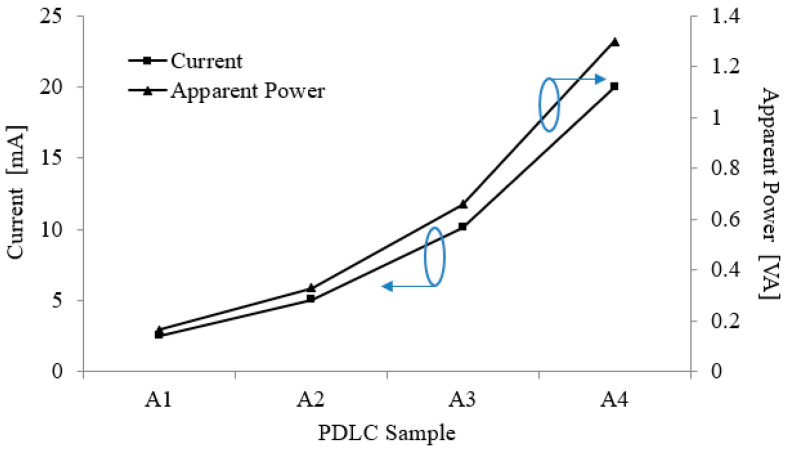
Current and apparent power consumption measurement for PDLC films with area ranging from 210 mm × 75 mm to 420 mm × 297 mm. The blue circles indicate their corresponding *y*-axes.

Two measurement sets were performed to determine the different PDLC film area effects on the film’s performance, which recorded the current and apparent power consumptions. Figure 5 depicts the increasing impact of the PDLC film area on the film’s power consumption. The labels A1, A2, A3, and A4 on the graph represent PDLC films with areas of 210 mm × 75 mm, 210 mm × 150 mm, 210 mm × 297 mm, and 420 mm × 297 mm, respectively. The circles on the graphs point toward their respective *y*-axis. According to the graph trend, as the area of the PDLC film doubled, the power consumption also doubled. The lowest current consumption measured was 2.53 mA at 210 mm × 75 mm, while the highest recorded current consumption was 20.0 mA at 420 mm × 297 mm. For a PDLC film to operate, an alternating current power supply is required [43]. This alternating current is responsible for reorienting the liquid crystals within the PDLC film. If the area of the film increases, the size of the liquid crystals inside the PDLC become larger. Hence, a higher alternating current amount is needed to reorient the liquid crystals to match the refractive index of the polymer matrix.

Similarly, the apparent power consumption of the PDLC films was investigated as the film area increased. When the area of the film increased, so did the apparent power consumption of the films. The minimum and maximum apparent power consumption values were 0.16 VA for 210 mm × 75 mm and 1.3 VA for 420 mm × 297 mm, respectively. Moreover, the graph revealed a trend similar to the area effect on the film’s current consumption. Based on Equation (1) above, this observation was due to the apparent power consumption of the PDLC film influenced by two parameters, namely the film’s input voltage and current consumption [29]. The current and apparent power consumptions were proportional since the input voltage was kept constant at 65 V throughout the experiment. Therefore, as the PDLC film area increased, the current drawn by the film also increased, which in turn caused the apparent power consumption to increase.

**Figure 6:**
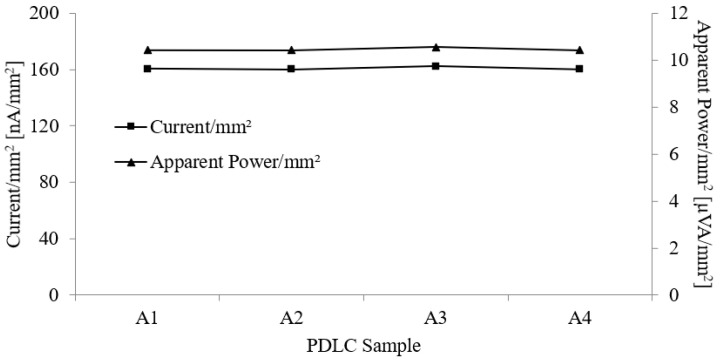
Current/mm^2^ and apparent power/mm^2^ measurement for PDLC films with area ranging from 210 mm × 75 mm to 420 mm × 297 mm.

Figure 6 shows the current/mm^2^ and apparent power/mm^2^ of different sizes of PDLC film. The current/mm^2^ recorded was approximately 160 nA/mm^2^, while the apparent power/mm^2^ value was 10.4 µVA/mm^2^. Based on the results obtained, it can be observed that these values remain constant despite the change in the area of the PDLC films. This is because the current and apparent power of the PDLC films increase linearly with larger sizes of the film. Hence, if the area of the film is doubled, the current and apparent power doubles too.

### 4.3. Varying Input Voltage Influence on the Light Transmittance of PDLC Films

**Figure 7:**
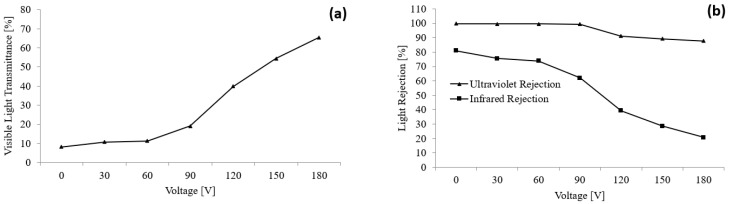
(**a**) Visible light transmittance and (**b**) ultraviolet and infrared rejection measurements with varying input voltage.

A PDLC film with a thickness of 0.394 mm and an area of 210 mm × 297 mm was applied in this study by supplying a varying input voltage. Consequently, a change in voltage caused the opacity of the PDLC film to alter, affecting the VLT, UVR, and IRR values (see Figure 7). Based on the graph, as the input voltage increased, the VLT transmittance value increased. The lowest recorded VLT was 8.2% at 0 V, while the highest VLT value was 65.5% at 180 V. Meanwhile, an increase in the voltage caused a significant drop in the IRR value. The largest IRR value was measured as 80.9% at 0 V, whereas the smallest IRR value was 20.7% at 180 V. Furthermore, a slight drop in UVR was noticed with an increasing input voltage. The highest recorded UVR percentage was 99.7% at 0 V, while 87.7% was the lowest measured IRR at 180 V.

The relation between light transmittance value and applied voltage can be linked using the following formula [44]:

(2) $$E_{loc\;}=\;\frac{V}{d}\left(\frac{3\rho_{LC}}{2\rho_{LC}+\rho_{\rho }}\right)$$

(3) $$T\propto E_{eff}=\;\left\lfloor E_{loc}+E_{int}\right\rfloor$$

where *E_loc_* is the electric field, *V* is the applied voltage, *d* is the film thickness, $\rho_{\rho }$ and $\rho_{LC}$ are the polymer and LC resistivities, respectively, *T* is the transmittance, *E_eff_* is the effective electric field, and *E_int_* is the electric field formed by ions [44]. Based on the formula, if the applied voltage increases, the electric field will increase too, causing the liquid crystals to align towards the electric field *E_eff_*; hence, the light transmittance value would increase [44]. As a higher percentage of light is penetrating through the PDLC film, the light rejection value would decrease, causing the UVR and IRR values to drop with higher applied voltage [44].

The increase in VLT with a higher applied voltage can also be explained with the orientation of the LC droplets. The LC droplets were randomly arranged at lower driving voltage as there was insufficient voltage. Therefore, only a smaller percentage of visible light could pass through the PDLC film [45]. In contrast, the LC droplets possessed higher voltage to reorient themselves in the direction of the electric field as the voltage was increased. This process caused the incident light to penetrate easily, causing the visible light transmittance percentage to rise. Alternatively, increasing the applied voltage minimizes the decrease in the UVR value [46]. This observation was owed to the cut-off wavelength of the PDLC film falling in the UV range (most of the incident UV light was blocked by the film). The IRR value decreased significantly with increasing voltage since the LC droplets could rearrange at higher input voltage in the electric field direction. Thus, a higher percentage of IR light penetrated the PDLC film causing the IRR value to drop.

Although the transmittance of the PDLC film was larger at higher voltage levels, the PDLC film was not advisable to operate at such a high voltage. This concern was because the optimum voltage for PDLC film to function is at its threshold [11]. Utilizing the PDLC film above its threshold voltage could cause the film’s lifetime to reduce, and the risk of damaging the film becomes higher. Applying a higher input voltage would also cause the apparent power consumption of the PDLC film to increase.

## 5. Conclusions

The sizing effects on the performance of the PDLC film were successfully investigated in this study. Based on the results obtained from conducting the experiment, it was evident that the transmittance of the PDLC films was affected by the size of the LC microdroplets, while different dimensions of the films caused changes in the current and apparent power consumptions of the films. Measurements for VLT demonstrated reduced transmittance with increasing film thickness due to more scattering of the incident light caused by larger LC droplets. Despite increasing the thickness of the PDLC film, the UVR percentage remained close to 100%, owing to the film’s cut-off wavelength in the UV range. Thicker PDLC films also observed an increase in the IRR percentage due to the larger liquid crystal droplets within them, which caused a higher percentage of incident light to bounce off during light penetration. In addition, the light intensity dropped with thicker PDLC films due to the scattering effect. Meanwhile, the film’s current and apparent power consumptions increased with a larger film size due to the realignment of large LC droplets within the PDLC film. It was concluded that larger PDLC films had bigger LC droplets; hence, more power was needed to change the LC droplets orientation.

A thinner PDLC film in this study was more cost-effective to adopt. There was no significant improvement in the film’s performance as the thickness increased. A 0.1 mm thick PDLC film was preferable to implement into various applications as the film’s performance and cost were substantially lower. Although the film’s current and apparent power consumptions increased with a larger area, the variations in the values were insignificant. In the working state, the largest PDLC film consumed only 1.3 VA. This power consumption was considered very minimal compared to the power required by other electrical appliances in homes and buildings. Therefore, the optimum dimension of PDLC films for large-scale deployment in commercial and building premises was 0.1 mm thickness with an area depending on the application. The light transmittance of the PDLC film was also affected by varying the applied voltage to the film. As the voltage value increased, the VLT increased too. Nonetheless, the IRR and UVR values dropped due to the voltage the LC droplets used to reorient themselves.

## Figures and Tables

**Table 1:** Summary of the dimension sizes of various PDLC films.

Sample	PDLC Film	
	Area (mm^2^)	Thickness (mm)
1	210 × 297	0.100
2	210 × 075	0.394
3	210 × 150	0.394
4	210 × 297	0.394
5	420 × 297	0.394
6	210 × 150	0.400
7	210 × 297	0.500

**Table 2:** Performance of PDLC films for different dimensions of the film.

Sample	PDLC Film									
	Area (mm^2^)	Thickness (mm)	VLT (%)		UVR (%)		IRR (%)		Light Intensity (lux)	
			OFF	ON	OFF	ON	OFF	ON	OFF	ON
**1**	210 × 297	0.100	10.2	81.2	100	99.7	82.1	12.1	637	756
**2**	210 × 075	0.394	9.7	80.6	99.8	62.3	78.2	13.9	626	769
**3**	210 × 150	0.394	9.7	80.6	99.8	63.5	78.2	13.9	626	769
**4**	210 × 297	0.394	9.7	80.6	99.8	62.1	78.2	13.9	626	769
**5**	420 × 297	0.394	9.7	80.6	99.8	62.8	78.2	13.9	626	769
**6**	210 × 150	0.400	9.7	77.2	100	99.6	83.5	16.6	624	749
**7**	210 × 297	0.500	6	73.7	100	99.8	89.2	17.6	558	726

## Data Availability

The data that support the findings of this study are available on request from the corresponding author.
